# The North Wyke Farm Platform: effect of temperate grassland farming systems on soil moisture contents, runoff and associated water quality dynamics

**DOI:** 10.1111/ejss.12350

**Published:** 2016-06-29

**Authors:** R. J. Orr, P. J. Murray, C. J. Eyles, M. S. A. Blackwell, L. M. Cardenas, A. L. Collins, J. A. J. Dungait, K. W. T. Goulding, B. A. Griffith, S. J. Gurr, P. Harris, J. M. B. Hawkins, T. H. Misselbrook, C. Rawlings, A. Shepherd, H. Sint, T. Takahashi, K. N. Tozer, A.P. Whitmore, L. Wu, M. R. F. Lee

**Affiliations:** ^1^ Rothamsted ResearchNorth WykeOkehamptonEX20 2SB DevonUK; ^2^ College of Life and Environmental SciencesUniversity of ExeterExeterEX4 4RJ DevonUK; ^3^ Rothamsted Research, Sustainable Soils and Grassland SystemsHarpendenAL5 2JQ HertfordshireUK; ^4^ AgResearch, Ruakura Research Centre, Farm Systems NorthPrivate Bag 3123Hamilton 3214New Zealand; ^5^ School of Veterinary SciencesUniversity of BristolLangfordBS40 5DU SomersetUK

## Abstract

The North Wyke Farm Platform was established as a United Kingdom national capability for collaborative research, training and knowledge exchange in agro‐environmental sciences. Its remit is to research agricultural productivity and ecosystem responses to different management practices for beef and sheep production in lowland grasslands. A system based on permanent pasture was implemented on three 21‐ha farmlets to obtain baseline data on hydrology, nutrient cycling and productivity for 2 years. Since then two farmlets have been modified by either (i) planned reseeding with grasses that have been bred for enhanced sugar content or deep‐rooting traits or (ii) sowing grass and legume mixtures to reduce nitrogen fertilizer inputs. The quantities of nutrients that enter, cycle within and leave the farmlets were evaluated with data recorded from sensor technologies coupled with more traditional field study methods. We demonstrate the potential of the farm platform approach with a case study in which we investigate the effects of the weather, field topography and farm management activity on surface runoff and associated pollutant or nutrient loss from soil. We have the opportunity to do a full nutrient cycling analysis, taking account of nutrient transformations in soil, and flows to water and losses to air. The NWFP monitoring system is unique in both scale and scope for a managed land‐based capability that brings together several technologies that allow the effect of temperate grassland farming systems on soil moisture levels, runoff and associated water quality dynamics to be studied in detail.

**Highlights:**

- Can meat production systems be developed that are productive yet minimize losses to the environment?
- The data are from an intensively instrumented capability, which is globally unique and topical.
- We use sensing technologies and surveys to show the effect of pasture renewal on nutrient losses.
- Platforms provide evidence of the effect of meteorology, topography and farm activity on nutrient loss.

## Introduction

Globally, civilization faces challenges in relation to land use and food production because of extremes in water supply (drought or flood), land degradation (urbanization or soil erosion), volatility in political conditions, spiralling energy costs, fluctuations in climate and threats from pests and pathogens (Bebber *et al.*, 2013). Grassland (pasture and rough grazing) is the largest crop by area in the UK, covering just over half of the entire landmass. In 2014 it accounted for 67% (12.35 M ha) of the total agricultural area, of which 89% was permanent grass or rough grazing (Defra, 2015). The forage it provides supports approximately 10 million cattle and 34 million sheep, with a net worth to the UK economy of around £8 billion per annum.

These grasslands also provide several other key ecosystem services that include support (e.g. water, nutrient cycling and soil protection), regulation (e.g. climate), culture (e.g. recreation) and bio‐control (e.g. source of predatory organisms). In spite of the importance of these services, little attention has been given to agricultural research at the farm scale. Most research has focused on replicated experimental plots or has been at the field scale. Pilgrim *et al.* (2010) reviewed the relations that exist in temperate grassland systems between agricultural production and ecosystem services. Eight of these services (climate, air quality, water quality, hydrological and soil erosion regulation, and nutrient cycling, biodiversity conservation and landscape quality) were examined by evaluating pair‐wise interactions. These authors concluded that negative relations arose only where the intensity of agricultural production increased, highlighting the need for the development of management strategies to reduce the effect of agricultural production on grasslands. In a subsequent review, Firbank *et al.* (2012) examined the trends in multiple ecosystem services and suggested that effective delivery of these services requires an improved understanding of how they are generated, their economics (developing markets) and their governance (regulation for environmental protection). Both these reviews suggest that food production should be integrated with the delivery of other ecosystem services by promoting a diversity of farming systems and the allocation of land use according to its suitability.

Previously in the UK, some studies have used farmlet approaches at the paddock scale (1 ha) when grazed by beef cattle in hydrologically isolated fields (e.g. Tyson *et al.*, 1992). Scholefield *et al.* (1993) monitored drainage water at V‐notch weirs and concluded that managing the fertilizer nitrate supply to the soil can reduce the leaching of nitrate. They also noted that the route of water movement through the soil to the watercourse determines the maximum nitrate concentration for a given load (N input). Laws *et al.* (2000) used the same paddocks as Scholefield *et al.* (1993) in a whole‐system approach with cut and grazed swards to investigate the effects of contrasting N inputs and management strategies for beef cattle on N budgets and herbage and animal production. The Cicerone project (e.g. Scott *et al.*, 2013) in Australia also used a farmlet approach to compare three unreplicated whole‐farm management systems at a scale considered to be credible to both livestock producers and researchers. Until recently there have been few research facilities in the UK to test hypotheses on sustainable agricultural production at a scale relevant to decision‐making by farmers (i.e. the farm scale) (McGonigle *et al.*, 2014). Although there are large‐scale ecological observatory (e.g. www.neoninc.org) and data observation (www.dataone.org) networks, the lack of facilities to monitor the effects of farm‐scale land‐management decisions has hindered our progress towards the development of more sustainable approaches to agricultural intensification. This has been resolved with the development of the North Wyke Farm Platform (NWFP) in an area of southwest England typical of lowland grassland systems in the UK. The NWFP is designated as a UK National Capability for collaborative research, training and knowledge exchange in agro‐environmental sciences, to address agricultural productivity and ecosystem responses to different management practices for temperate grassland.

This paper describes the establishment of the NWFP and the various datasets that have been, are being or will be collected to achieve key research objectives. The data obtained from the NWFP are publicly available (http://www.rothamsted.ac.uk/farmplatform). We also describe a case study that covers the 3‐month period of September to November 2013 and investigated the response of soil moisture levels and runoff as a function of the weather. This demonstrates that farm management is instrumental in determining runoff and associated water quality dynamics. The case study illustrates the potential of the NWFP to assess quantitatively the effectiveness of farm management strategies with a wide range of criteria and the power of continuous monitoring to measure effects on the environment. Finally, we place the NWFP in the broader context of advancing research into sustainable pastoral agriculture and, in particular, understanding soil responses and water discharge.

## Materials and methods

### Set‐up and background

The infrastructure of the NWFP farm‐scale experimental system was established in 2010 on the North Wyke Farm in the southwest of England (50°46′10″N, 3°54′05″W); it is described in detail by Orr *et al.* (2011). The NWFP comprises three individual farmlets, each of approximately 21 ha, designed to test the productivity and environmental sustainability of contrasting temperate grassland beef cattle and sheep systems. Each of the three farmlets consists of five catchments that range in size from 1.62 to 8.08 ha. Each catchment is hydrologically isolated through a combination of topography and a network of 9.2 km of French drains (800‐mm deep trenches that contain a perforated drainage pipe backfilled to the surface with 20–50 mm clean granite, carbonate‐free, stone chips) that was constructed at the edges of the catchments. Each catchment is equipped with monitoring sites for rainfall, soil moisture, discharge and water physicochemical properties. There is also a single site for the collection of meteorological data. Six of the 15 catchments have field divisions providing 21 fields in total across the NWFP. (See Figure S1 in Supporting Information for a map of the farmlets).

**Figure 1:**
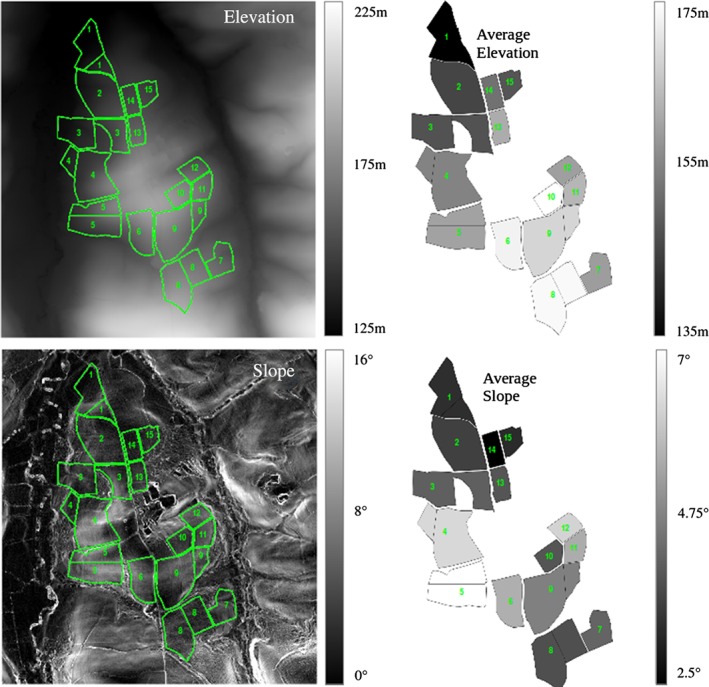
The North Wyke Farm Platform showing the field layout overlaid on satellite elevation and slope images of the site, together with the average elevation and average slope of each catchment.

The NWFP is on a ridge at 120–180 m above sea level; the land slopes to the west to the River Taw and to the east to one of its tributaries, the Cocktree stream. A digital surface model (DSM) and digital terrain model (DTM, Figure 1) have been produced from LiDAR (light detecting and ranging) data (Ferraccioli *et al.*, 2014). The soil (Harrod & Hogan, 2008) belongs predominantly to two similar series, Hallsworth (Dystric Gleysol) and Halstow (Gleyic Cambisol) (Avery, 1980), which comprise a slightly stony clay loam topsoil (approximately 36% clay) that overlies a mottled stony clay (approximately 60% clay), derived from underlying Carboniferous culm rocks. Below the topsoil layer, the subsoil is impermeable to water and is seasonally waterlogged; most excess water moves by surface and sub‐surface lateral flow across the clay layer to be intercepted by the bounding drainage system at the edge of each catchment. (See Figure S2 in Supporting Information for a map of the principal soil series).

From 1984 to 2013, the mean and median annual precipitation at North Wyke was 1040 and 1031 mm, respectively. This had a distribution with an interquartile range from 922 to 1146 mm. Over this 30‐year period, the distribution of the minimum daily temperatures had an interquartile range from 3.4 to 10.2°C, whereas the distribution of the maximum daily temperatures had an interquartile range from 9.6 to 17.2°C. North Wyke has a large and consistent amount of rain in summer, which is characteristic of the major agricultural grassland areas in the west of the UK.

### Farmlet management and treatments

Over a 2‐year period, from 1 April 2011 to 31 March 2013, the beef cattle and sheep systems operated under the same management guidelines on all three farmlets to enable productivity to be measured on the existing permanent grassland. From 1 April 2013, two of the farmlets entered a ‘transition’ phase, where they moved progressively towards the following treatments:

1. *Blue farmlet, legumes*. Sward was improved by reseeding with long‐term grass (perennial ryegrass; *Lolium perenne* L.) and legume (white clover; *Trifolium repens* L.) mixtures. Clover‐based systems can replace (Elgersma & Hassink, 1997) up to 150 kg nitrogen (N) ha^−1^ of industrially‐produced N, which is a major cost for any grassland farm. An opportunity to reduce costs might lie in farmers' becoming more reliant on N that is biologically fixed by legumes. On this farmlet, we enhanced the current small proportion of clover by reseeding, but we do not rely on clover alone to supply the N. In addition, a maximum of 40 kg N ha^−1^ of fertilizer is permitted in spring in a particularly cold, slow growing season, and organic manures are also used. However, no inorganic N fertilizer was required in the period 2013–2015 on the reseeded fields.
2. *Red farmlet, planned reseeding*. Sward improvement through planned and regular reseeding about every 4 years. Currently, new varieties developed by plant breeders for traits associated with improved animal performance (e.g. grasses with enhanced sugar content; polymeric‐oligo‐fructans) or environmental resilience (e.g. deep‐rooting grasses) have been sown on this farmlet. In the future, this treatment will provide opportunities to introduce other new and innovative plant variety traits (e.g. grasses with large lipid content, clover with large polyphenol oxidase content, clover with small protein content) developed to improve the efficiency of nutrient use by the animals and greater environmental resilience that can be incorporated easily.
  The progressive reseeding was achieved over 3 years and was intended to allow the systems to continue to run and feed the cattle and sheep. The Blue and Red treatments will be compared with the following Green control.
3. *Green farmlet, permanent pasture*. Sward improvement of the existing grassland through the use of artificial fertilizers, and with monitoring of the proportion of original sown species (predominantly perennial ryegrass). Both the Red and Green farmlets are fertilized with nitrogenous fertilizer (see below).

Individual catchments within the Red and Blue farmlets were sprayed with glyphosate to kill the existing grass, followed by ploughing and cultivation, and then reseeded in July to August 2013, 2014 and 2015 (0.40, 0.34 and 0.26 of the farmlets were reseeded in each year, respectively). (See Figure S3 in Supporting Information for a map of the re‐seeding schedule).

### Livestock

Each farmlet on the NWFP was grazed by yearling beef cattle (25 in 2011, 27 in 2012 and 30 between 2013 and 2015) and ewes (50 between 2011 and 2014 and 75 in 2015) and their lambs. (See Figure S4 in Supporting Information for a generalized management plan).

### Grazing management

The fields were continuously stocked with cattle and sheep (Allen *et al.*, 2011). Fields are grazed by cattle or sheep, or set aside for silage if not required for grazing following typical practise.

The combination of fertilizer application with the nutrient inputs from grazing livestock provides an opportunity to study the effect of animals on soil physical, biological and chemical properties. A heterogeneous distribution of N from urine and dung deposition, soil compaction and poaching, result from the animals, together with nutrient cycling from inorganic and organic sources. These will also affect water flows and chemical composition as well as microbiology, for example from faecal material. Losses to the atmosphere, particularly as greenhouse gases, will also be affected by this management. In addition, applications of manure to soil cause N flushes and affect soil carbon and P.

### Inorganic and organic fertilizers

The fertilizer rates used each year followed the UK ‘Fertilizer Manual (RB209)’ guidelines (Defra, 2010). Both the Red and Green farmlets are fertilized with up to 200 kg N per ha of nitrogenous fertilizer. All three treatments are fertilized with P, K and S before cutting and also when the values from soil analyses are below target values (Soil Index 2 for P and 2– for K), and lime is applied when the pH is below 6 for grassland.

The cattle and sheep are housed and bedded in winter on purchased barley straw; therefore, the farmyard manure (FYM) produced is analysed for nutrient content and applied to fields due to be grazed following the cutting of silage. The exact fertilizer and FYM spreading areas for each field are determined with GPS and ArcGIS^TM^ tools, and account is taken of UK regulatory (Nitrate Vulnerable Zone Action Programme rules; Statutory Instrument 668‐2015; Defra, 2013) requirements to avoid applications within 2 m (inorganic fertilizer) or 10 m (organic manure; 6 m if precision spreading equipment is used) of watercourses.

### Continuous monitoring with instrumentation and telemetry

The systems and sensors used to monitor soil and water on the NWFP have been described in detail by Griffith *et al.* (2013). A wireless UHF radio telemetry network consists of: (i) remote telemetry units (RTUs) that record data from instruments in the field and transmit by UHF radio, (ii) a centrally‐located base‐station that manages the network and (iii) software to record, store, process and display the data by an integrated web server. Currently (November 2015), the NWFP has a network of 47 RTUs connected to 110 instruments that record data on 200 environmental variables every 15 minutes. A fibre optic network has also been installed to provide data connectivity to all 15 flume laboratories.

### Meteorological stations

Two sets of meteorological instruments are co‐located at a central meteorological station: (i) an official UK Meteorological Office site and (ii) a site specific to the NWFP experiment, with data recorded since April 2013. For the latter, the following meteorological variables are recorded: precipitation from the rain gauge, air temperature, relative humidity, wind speed, wind direction and solar radiation (installed in May 2014). The NWFP meteorological data are recorded at 15‐minute intervals.

### Soil moisture stations and rain gauges

A soil moisture station (SMS) is located approximately centrally within each of the 15 catchments and measures soil moisture (%) through capacitance at depths of 10, 20 and 30 cm and soil temperature at 15 cm. There is also a tipping‐bucket rain gauge.

### Flow measurement and sampling non‐continuous flow with a bypass cell

The quantity of runoff from each catchment is measured through a combination of H‐flumes and bubbler flow‐meter devices. Two issues associated with the measurement of agricultural runoff at the field scale are that flow is not continuous, but rather linked to soil moisture conditions and rainfall events, and the sensors used are vulnerable to drying out and must remain wet at all times. The NWFP system deals with this by pumping water automatically every 15 minutes into a stainless steel bypass cell where the sensing of water quality variables occurs when flow conditions allow, but retains the previous sample when the flow rate is below a threshold. This is achieved through a combination of the data from the flow‐meter, the telemetry network, a programmable logic controller and a bidirectional peristaltic pump.

### Flume laboratories

Each of the 15 catchments drains to a single monitoring station supplied by two branches of each French drain system and these join in a confluence pit. The water then flows first to a pre‐collection chamber (which provides access for the collection of samples) and then to an open channel flow nozzle with free‐falling discharge conditions. Each site has a flume laboratory that houses pumping equipment, a bypass cell, telemetry devices and sensors to measure the physical and chemical properties of water. Data collected from all 15 catchments include: discharge, nitrate and nitrite, dissolved organic carbon, ammonia, ammonium, conductivity, dissolved oxygen, pH, temperature and turbidity. Total phosphorus (total P) and orthophosphate (ortho P) are measured at three catchments (numbers 2, 5 and 8), one in each farmlet (Figure 1). Total P and ortho P are measured on a campaign basis (short periods of intensive measurements) because the instruments need to be switched on manually and shut down according to flow conditions.

Each flume laboratory also contains an online auto‐sampler for the unattended regular collection of water samples. These devices are connected to the telemetry network so that they can be triggered remotely or set to trigger proportionally with other variables in the system. Typically this is flow, with the samplers set to collect samples on the rising and falling limbs of a storm hydrograph. The collection of physical samples enables the analysis of variables that are not being measured continuously as part of the system and gives great flexibility in measuring an array of water contaminants that include macro‐ and micro‐minerals, chemical residues and biological material such as faecal indicator organisms.

### Field surveys

The NWFP fields were surveyed to collect ‘baseline’ data for: (i) plant nutrients in July 2012 (Shepherd *et al.*, 2014) and (ii) plant species' abundance in July to August 2013. The soil survey measured seven variables: bulk density, total carbon, total N, soil organic matter, pH, *δ* ^13^C and *δ* ^15^N. The botanical survey used the DOMIN method (Rodwell, 1992), and 18 different species were identified. The permanent pastures contained on average 64% *Lolium perenne*, 38% *Agrostis stolonifera*, 2% *Holcus lanatus* and 1% *Alopecurus geniculatus* as the main constituents. Because these baseline field surveys were carried out in different years (a consequence of available resources and survey practicalities) the 2012 soil data were supplemented by smaller surveys (usually three or four catchments) in 2013, 2014 and 2015. Future surveys will be designed and implemented to be spatially and temporally coherent, and will be managed according to the statistical model outputs of the above ‘baseline’ survey datasets (Wang *et al.*, 2012; Webster & Lark, 2013).

### Data management and utility

The NWFP monitoring system is unique in both scale and scope for a managed land‐based capability. It brings together several technologies that enable the effect of temperate grassland farming systems to be studied in depth. Rigorous data management, quality control and validation provide the basis for accurate assessments of the losses and gains between increased agricultural production and the provision of ecosystem services, at any given time interval, for each of the three treatments. Like the Cicerone project in Australia, the NWFP is investigating unreplicated whole‐farm management systems with equivalent starting conditions at a scale that is credible to livestock producers and land managers.

## Results and discussion

### Case study

The period September to November 2013 was chosen as a case study to illustrate the utility of the NWFP datasets. This represented a period of ‘typical’ meteorological activity, with no atypical extreme weather events, which facilitates a reasonably straightforward interpretation of the data. Crucially, this period coincided with the first round of reseeding, where four of the catchments (2, 8, 14 and 15) were ploughed, cultivated and reseeded in July 2013. These disturbances, together with the topography and soil type, were likely to affect the hydrology and nutrient runoff dynamics.

**Figure 2:**
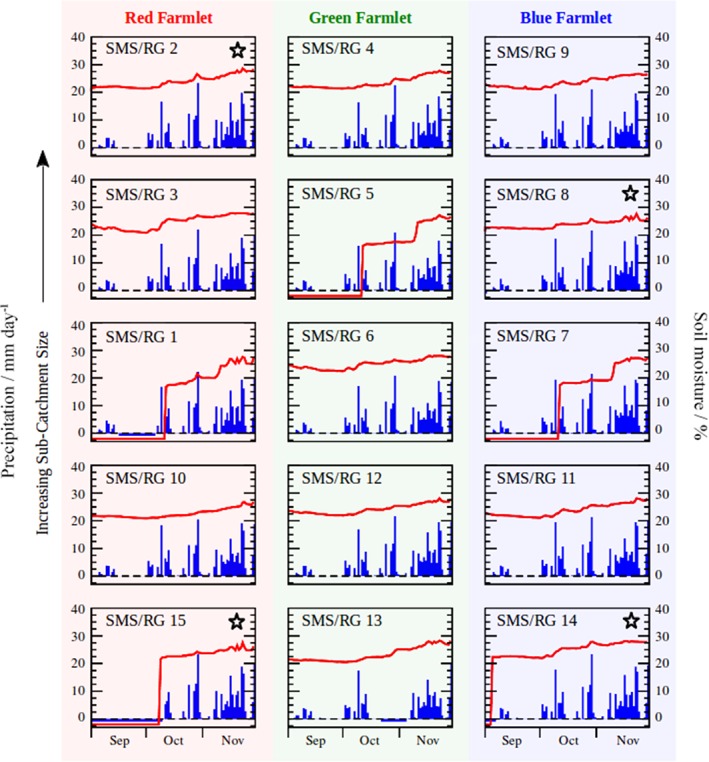
Recorded precipitation (blue bars; mm day^−1^) and soil moisture (averaged for values measured at depths of 10 and 20 cm; red solid line; %) in each of the catchments during the period September to November 2013. SMS, soil moisture station; RG, rain gauge; 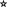,ploughed catchment.

A selection of the data collected by the rain gauges and SMSs during this period is summarized in Figure 2, including the amount of precipitation (blue bars) and soil moisture content (red lines); the latter was averaged for values measured at depths of 10 and 20 cm. The 15 graphs (one for each catchment) show average data over each individual day, which makes the graphical depiction of the data substantially clearer and easier to interpret because the 15‐minute interval data include many large and rapid short‐term fluctuations. A similar pattern of particularly intense daily rainfall totals can be seen. The soil moisture readings provide valuable information on the degree of saturation of the soil, which affects its potential for sustaining plant growth, risk of compaction and damage from livestock poaching, and on its capacity to absorb more water during further precipitation events.

Missing data points correspond to periods when a sensor was either not functioning or it returned unreliable values. Care is taken to ensure that periods of missing precipitation data are not mistaken for dry periods (i.e. missing data should not be assigned ‘false zero’ readings). Because all rain gauges are in relatively close spatial proximity, a dry day will almost always result in zero precipitation readings at all rain gauges. Thus ‘false zero’ readings can be identified by their temporal overlap with non‐zero precipitation readings on other nearby functional rain gauges. Missing precipitation data are given negative values (−1) to avoid confusion in Figure 2. There were distinct periods of missing precipitation data in catchments 1, 13 and 15. Similarly, periods of missing soil moisture data are also given as negative values (−2) in Figure 2, and such periods occurred in catchments 1, 5, 7, 14 and 15 at the beginning of the case study period (relating to a period when malfunctioning soil moisture sensors were replaced).

**Figure 3:**
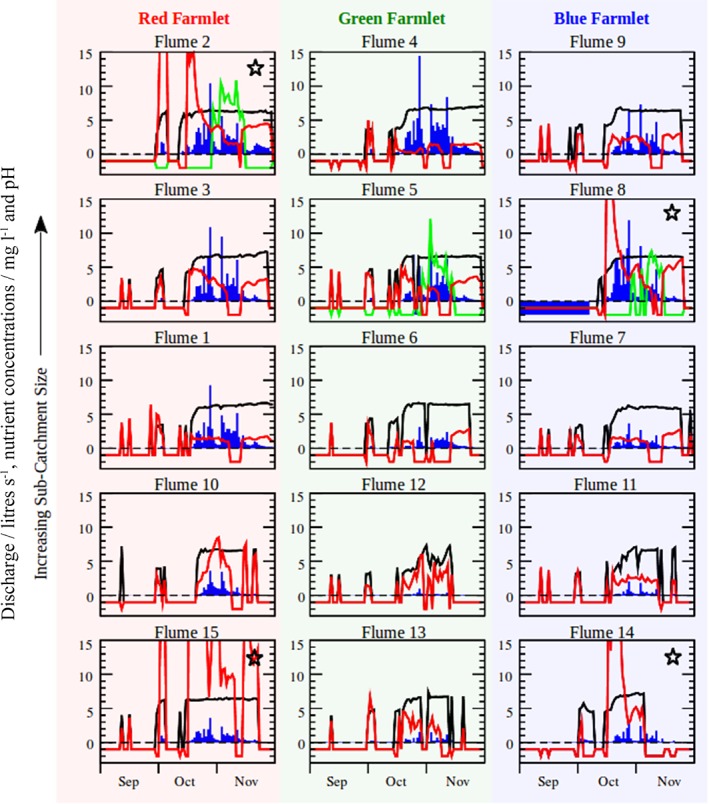
Recorded discharge (blue bars; litres s^−1^), total P concentration (green solid lines; mg l^**−**1^), total N concentration (red solid lines; mg l^**−**1^) and pH (black solid lines) at each of the flume laboratories on the North Wyke Farm Platform during the period August to November 2013. Phosphorus concentrations are only measured at flumes 2, 5 and 8. 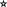, ploughed catchment.

Figure 3 shows a selection of the data acquired by the flume laboratories during this period, including discharge, total inorganic N (ammonium plus nitrate and nitrite), total P and pH. Again, the 15‐minute resolution of the data has been averaged over each day to smooth out the large oscillations that complicate visual interpretation. If the flow is too small (i.e. rates of less than 0.2 l s^−1^) to record concentrations (total P excluded) then the corresponding missing water chemistry data (i.e. total inorganic N and pH, in this case) are assigned a value of −1. If a given sensor is not functioning correctly (including the flow sensor itself), however, then the corresponding missing data are assigned a value of −2. From Figure 3, it is clear that missing data will be a key component of these data because little or no data are expected during dry periods.

**Figure 4:**
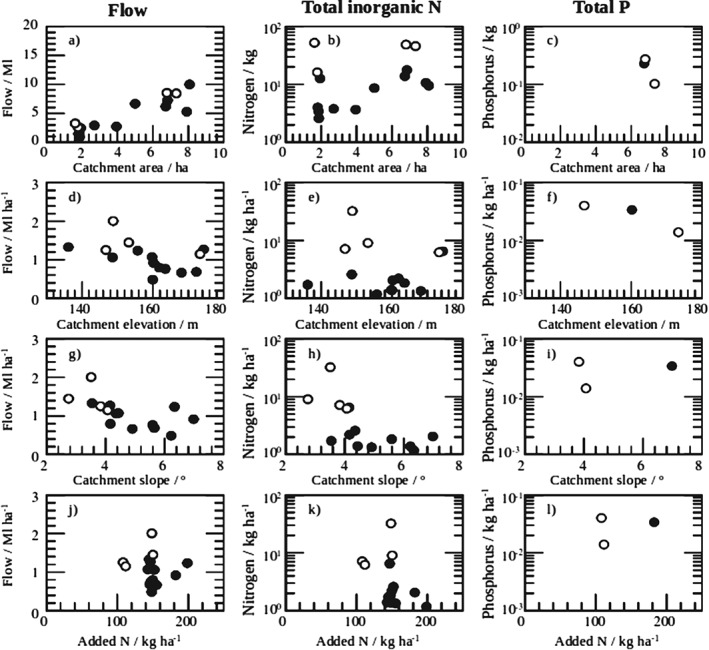
Total accumulated discharge (a, d, g, j), total inorganic nitrogen (b, e, h, k) and total phosphorus (c, f, i, l) runoff accumulated by each catchment over the 3‐month study period. The data points are plotted against catchment area, average catchment elevation, average catchment slope and total amount of nitrogen (N) added as fertilizer. Open points denote catchments ploughed immediately prior to the case study period; solid points denote unploughed catchments.

A comparison of Figure 3 with Figure 2 shows that discharge (blue bars in Figure 3) has the expected hysteretic lag effect with precipitation (blue bars in Figure 2). Furthermore, the magnitude of the discharge (Figure 3) will not only depend on soil type and saturation, but also on the catchment size (see also Figure 4). The sharp peaks in total inorganic N concentrations in Figure 3 can be attributed to the first heavy rains of the season, which wash ions out of the soil and into the drains surrounding the catchments (cf. Jiang *et al.*, 2010; Yang *et al.*, 2015). Interestingly, these effects are most marked for the four catchments that were ploughed and reseeded (i.e. 2, 8, 14 and 15), whereas the remaining catchments display a more moderate response. Total P provides inconclusive results in this respect because it was measured in only three of the catchments. For pH, it should be noted that the pH readings are initially quite low (acidic) when the flume apparatus first switches on and they then level out to known soil pH values. The reasons for this are unclear, but they might be the result of some biological process that slowly acidifies the water content of the drains under zero flow conditions.

The data shown in Figure 3 were used to calculate the total volume of water accumulated by each catchment, and the total quantities of discharge and associated nutrients enable the economic and general environmental cost of the losses to water from each catchment to be assessed. In the long term, this information can be used to assess the cost‐effectiveness of the treatments for the three farmlets. These management schemes have not yet been fully implemented; therefore, we use the effects that catchment characteristics and farm operations can have on runoff dynamics as an example here. Figure 4 shows the integrated total quantities of discharge, and associated total inorganic N and total P runoff accumulated by each catchment over the 3‐month study period. The data points are plotted against catchment area, average catchment elevation, average catchment slope and total amount of N added as fertilizer. The total amount of water and nutrients accumulated by a catchment correlates broadly with its area, given in the top row of Figure 4 (and also indicated in Figure 3). Therefore, both the flow and the nutrient data are standardized by catchment area in the scatterplots given in the second, third and fourth rows.

The results of inorganic N show trends that are common to some of the flumes; for example, large concentrations may persist for long periods, as in flumes 2, 3, 10 and 15 in the Red farmlet. A relation with pH appears in some of the flumes; this is particularly evident in flumes 12 and 13. Total P measurements were restricted to a 2‐week period from 30 October to 15 November, but they are presented to indicate the type of data that can be acquired. In general, concentrations correlate positively with discharge, but with no discernible effects from ploughing compared with the unploughed catchment.

From the scatterplot matrix in Figure 4, it is clear that average elevation, average slope and total added N are not correlated with the flow of water or total amounts of inorganic N or total P. Catchment topography has little effect on the recorded runoff because each catchment is a closed system with no opportunity for runoff to flow from one catchment to another. Furthermore, we use topographic averages in this analysis, which is a gross simplification of the real topography. Rather surprisingly, concentrations of total inorganic N do not increase when more nitrogenous fertilizer is added; this result warrants further investigation. The most striking differences, however, occur for the flow of water and loads of total inorganic N between unploughed (solid points) and ploughed (open points) catchments (Figure 4), where flow and nitrogen loads tend to increase after ploughing. Similar patterns have been noted by previous research and attributed to the stimulation of N mineralization in conjunction with ploughing and reseeding (see Watson & Younie, 1995; Shepherd *et al.*, 2001). This simple analysis demonstrates that topsoil disturbance is a crucial factor in determining the quantity of mineral nutrient runoff in ‘first flush’ storm events. Such visual evidence was confirmed by an analysis of variance (anova) on the data in R (http://www.r‐project.org) with the standard analysis of variance model command aov(). We also included the soil series data because of their potential relations with flow and nutrient loss in runoff. The results show that the only significant predictors of flow (*P* = 0.013) and total inorganic N (*P* = 0.023) were whether the catchments were unploughed or ploughed (see Tables 1 and 2).

**Table 1:** Results of anova for total water flow per hectare

Source of variance	Degrees of freedom	Sum of squares	Mean square	*F*‐ratio	Probability (> *F*)
Soil type	4	4.118e + 11	1.030e + 11	1.560	0.2848
Ploughed or unploughed	1	7.682e + 11	7.682e + 11	11.64	**0.0113**
Catchment slope	1	9.262e + 10	9.262e + 10	1.403	0.2749
Total fertilizer added per hectare	1	3.039e + 10	3.039e + 10	4.603	0.0691
Residuals	7	4.621e + 11	6.602e + 10	—	—

Boldface indicates significance at 5% level.

**Table 2:** Results of anova for total inorganic N per hectare

Source of variance	Degrees of freedom	Sum of squares	Mean squares	*F*‐ratio	Probability (> *F*)
Soil type	4	1.165e + 14	2.912e + 13	0.611	0.668
Ploughed or unploughed	1	4.002e + 14	4.002e + 14	8.404	**0.023**
Catchment slope	1	4.651e + 12	4.651e + 12	0.098	0.764
Total fertilizer added per hectare	1	2.797e + 13	2.797e + 13	0.587	0.469
Residuals	7	3.334e + 14	4.763e + 13	—	—

Boldface indicates significance at 5% level.

In summary, this case study exemplifies a way in which water and nutrient runoff from the NWFP catchments can be monitored and linked to topography, soil and farming activity. Corresponding measurements for precipitation and soil moisture place such discharge and nutrient runoff data usefully into context, and when considered as a whole, the NWFP clearly provides a rich data resource for model calibration and application. Our case study was deliberately basic to provide an example of the utility of these data. The three management strategies of the NWFP came to maturity in late 2015; therefore, this type of investigation will enable a more complex and comprehensive assessment of the utility of each strategy to maximize farm productivity while minimizing any detrimental effects on ecosystem services. This will be especially true when more datasets of livestock production, relevant farm management and greenhouse gas (GHG) data, which are also being collected, are included.

### Current and future NWFP projects

The NWFP is currently being used to assess the effect on selected sustainability metrics (indicators of sustainability) of grasses bred to have enhanced concentrations of sugar (polymeric‐oligo fructan; water‐soluble carbohydrate) and deep‐rooting traits (see www.sureroot.uk), sown in monocultures or in mixtures with white clover. These metrics indicate, for example, the effect on soil health in terms of physical structure, nutrient status and microbiology. These grasses constitute key intervention strategies on the Red (planned reseeding) and Blue (legume) farmlets, which will be assessed over 3 years to ensure that the data are robust and will be treated as long‐term experiments. Once the effects of the current interventions have been assessed (2015–2018), the next aim will be to develop (together with partners from academia and industry) a suite of novel and practical interventions. The results from these developed interventions will need to be translated to the regional, national and global scales. Inclusion of the NWFP in initiatives such as the Global Farm Platform (GFP) (www.globalfarmplatform.org) will facilitate translation to effects in other grazing systems with different climates and soil types. The systems‐based approach of the NWFP, underpinned by robust data collection, will help to identify the most optimal livestock grazing systems for temperate grassland. Positive outcomes can be adopted subsequently by the farming community to ensure sustainable livestock production (in societal, environmental and economic terms), and in particular to make the best use of our soil to optimize the use of land for food production while protecting the environment, and to help direct and develop national and international agricultural policy. For example, current research at the NWFP is investigating the effect of changing management on soil carbon stocks.

Well‐managed permanent grasslands used for grazing cattle and sheep for beef and lamb production are already recognized as carbon sinks under appropriate forage management through the lack of cultivation and recycling of carbon and nutrients directly and indirectly through inputs of manure. The major threat to the sequestration of carbon by grazed grasslands is a potential change in land use to arable cultivation for food and fibre production. The environmental effects of these types of farming in relation to GHG emissions are described as being more benign than ruminant production, largely because of the release of methane (CH_4_) from ruminant enteric fermentation. Cultivation, however, unlike well‐managed permanent grasslands, causes soil degradation and the loss of soil carbon, both directly as CO_2_ and indirectly by erosion of vulnerable bare soil and the soil carbon bound to it on sloping land. Quantification of the effects of individual management practices and their combinations on carbon sequestration is, therefore, vital for improving the potential of farming systems to sequester carbon (Smith *et al.*, 2004). To understand why soil has a particular carbon content, it is necessary to quantify both inputs and outputs of carbon to the system. Unfortunately, these are both difficult to measure and the difference between input and output is usually small, which further complicates estimates of change in storage (Rees *et al.*, 2005) and leads to large uncertainties in the location of carbon sinks and their activity in temperate grasslands (Jones, 2010).

### Models, data analysis and precision or ‘smart’ agriculture

The NWFP provides the research community with a clear opportunity to develop, apply and evaluate farm‐scale models for grazing livestock systems and so provide predictive capability for a range of ecosystem services. Wu *et al.* (2015) used baseline datasets to model the dynamics of soil water content, water discharge and removal of forage biomass, and also investigated climate change scenarios. They concluded that reliable estimates could be made from these simulations. A priority is the development and testing of farm typology models to evaluate different scenarios, and at the more mechanistic level to model interactions between different species and functional groups in swards on a particular soil type. The dynamics of feed quality for different forages and forage mixtures is a critical area for development to extend the capability of the SPACSYS modelling system (Wu *et al.*, 2007). These models will also enable the effects of the above to be tested on different types of soil. Furthermore, the NWFP provides the means to develop broader assessments of the effects downstream of ‘business as usual practice’, changes in management system and the provision of full Life Cycle Assessment (LCA) metrics, including C, N, P, sediment or water footprints of beef and sheep farming systems. Further assessment of carcass and product quality (meat) can extend the LCA assessment to include potential anthropogenic implications such as health (e.g. mineral, vitamin and fatty acid composition of the meat). This will expand the comparison of food production systems (e.g. arable) away from simply product per hectare to delivery of nutrients per hectare and the associated effect of the environment on production. In addition, food production is relevant to human health, which has strong links with soil properties (Dungait *et al.*, 2012).

Opportunities also exist for the application and development of a wide range of statistical methodologies to the NWFP data; including the possibility of mechanistic and statistical model hybrids (e.g. Orton *et al.*, 2011; Clifford *et al.*, 2014) that examine the complexity of processes whilst at the same time account for data and model uncertainty. The NWFP also enables the development of biodiversity statistical models that indicate field‐scale distributions of pests, diseases and pathogens and how these distributions interact with livestock production. Natural heterogeneity in soil because of topography and parent material, livestock grazing behaviour and soil management will affect nutrient and moisture distribution, and also spatial differences in gaseous emissions, especially GHG, that can be studied with these statistical methods. Furthermore, there are many unique opportunities for up‐scaling the information obtained from the NWFP, such as following faecal indicator organisms in the environment, with the nearby River Taw as a test landscape.

Appropriate model development in tandem with appropriate (ground‐based and remote) sensor technologies can also provide opportunities to develop and assess precision and smart agriculture techniques on the NWFP for pasture‐based ruminant production systems. In this respect, the NWFP has the potential for research collaboration with partner institutions far removed from those focused on agriculture (e.g. computer scientists, sensor technologists, engineers, and so on).

### Design and refinement of the NWFP experiment

Methodologies for data collection on the NWFP are not static. For example, new sensors can be integrated easily or existing ones configured to measure different variables; data recording rates can be changed, equipment can be triggered when thresholds are reached, and this can all be visualized and controlled from a computer connected to the web. The UHF radio network has scope for the incorporation of many more sensors, and the inclusion of a fibre optic network in all 15 flume laboratories offers further opportunity for expansion with the emergence of new sensing technologies (Griffith *et al.*, 2013). Although the long‐term integrity of the data collection should be preserved as far as possible, continuous statistical review and assessment might suggest that some data have little scientific value and should no longer be obtained; conversely, some other types of data might be considered important that were not considered originally from the outset. Care should also be taken to ensure the coherence of the NWFP data so that data can be related in both space and time, and across all relevant scales. This is not always feasible because data collection is commonly constrained by available resources, the particular needs of the farm operations and the inherent unpredictability of the local climate.

## Conclusion

This rich data resource provides valuable and varied research opportunities to further our understanding of sustainable grazing livestock systems. We have described a simple case study of field discharge and associated nutrient dynamics to demonstrate the utility of this farm‐scale experiment by a quantitative assessment of the effect of meteorology, field topography and farming activity on soil nutrient loss, with a particular subset of the NWFP data. Careful management of the soil and plant resources can lead towards sustainable livestock production and produce many social, economic and environmental benefits. This will not only improve productivity (and potentially product quality), but also reduce the ecological footprint and generate a diversity of ecosystem services such as improved water, air and soil quality, all of which are critically important in this era of climate change and global food security.

## Supporting information


**Figure S1.** The North Wyke Farm Platform, with Red, Green and Blue farmlets (bounded at the edges by French drains) shown, together with the locations of the soil moisture stations, rain gauges (red circles) and flume laboratories (blue squares). The three flume laboratories that can record phosphorus levels are marked (green diamonds). The single on‐site meteorological station is marked with a black cross.
**Figure S2.** The North Wyke Farm Platform with the field boundaries overlaid on a map of the principal soil series, together with the 5‐m contour lines.
**Figure S3.** The North Wyke Farm Platform, with the re‐seeding schedule for the Red and Blue fields in 2013–2015 shown. Flume outlets are indicated (blue squares) together with the corresponding catchment numbers. The Green control fields remain undisturbed as long‐term permanent pastures.
**Figure S4.** The North Wyke Farm Platform with groups of fields (A, B1, B2, C, D, E and F) shown as ‘enterprise triplets’, which are planned to have similar management. Here we show a generalized plan for April to June.
