# Supplementary material for: The North Wyke Farm Platform: effect of temperate grassland farming systems on soil moisture contents, runoff and associated water quality dynamics
Source: Eur J Soil Sci. 2016 Jun 29;67(4):374–85. doi: 10.1111/ejss.12350 (PMC5103177; doi:10.1111/ejss.12350)
Supplement: Supplementary file 1 — Figure S1. The North Wyke Farm Platform, with Red, Green and Blue farmlets (bounded at the edges by French drains) shown, together with the locations of the soil moisture stations, rain gauges (red circles) and flume laboratories (blue squares). The three flume laboratories that can record phosphorus levels are marked (green diamonds). The single on‐site meteorological station is marked with a black cross. Figure S2. The North Wyke Farm Platform with the field boundaries overlaid on a map of the principal soil series, together with the 5‐m contour lines. Figure S3. The North Wyke Farm Platform, with the re‐seeding schedule for the Red and Blue fields in 2013–2015 shown. Flume outlets are indicated (blue squares) together with the corresponding catchment numbers. The Green control fields remain undisturbed as long‐term permanent pastures. Figure S4. The North Wyke Farm Platform with groups of fields (A, B1, B2, C, D, E and F) shown as ‘enterprise triplets’, which are planned to have similar management. Here we show a generalized plan for April to June. [file EJSS-67-374-s001.docx]

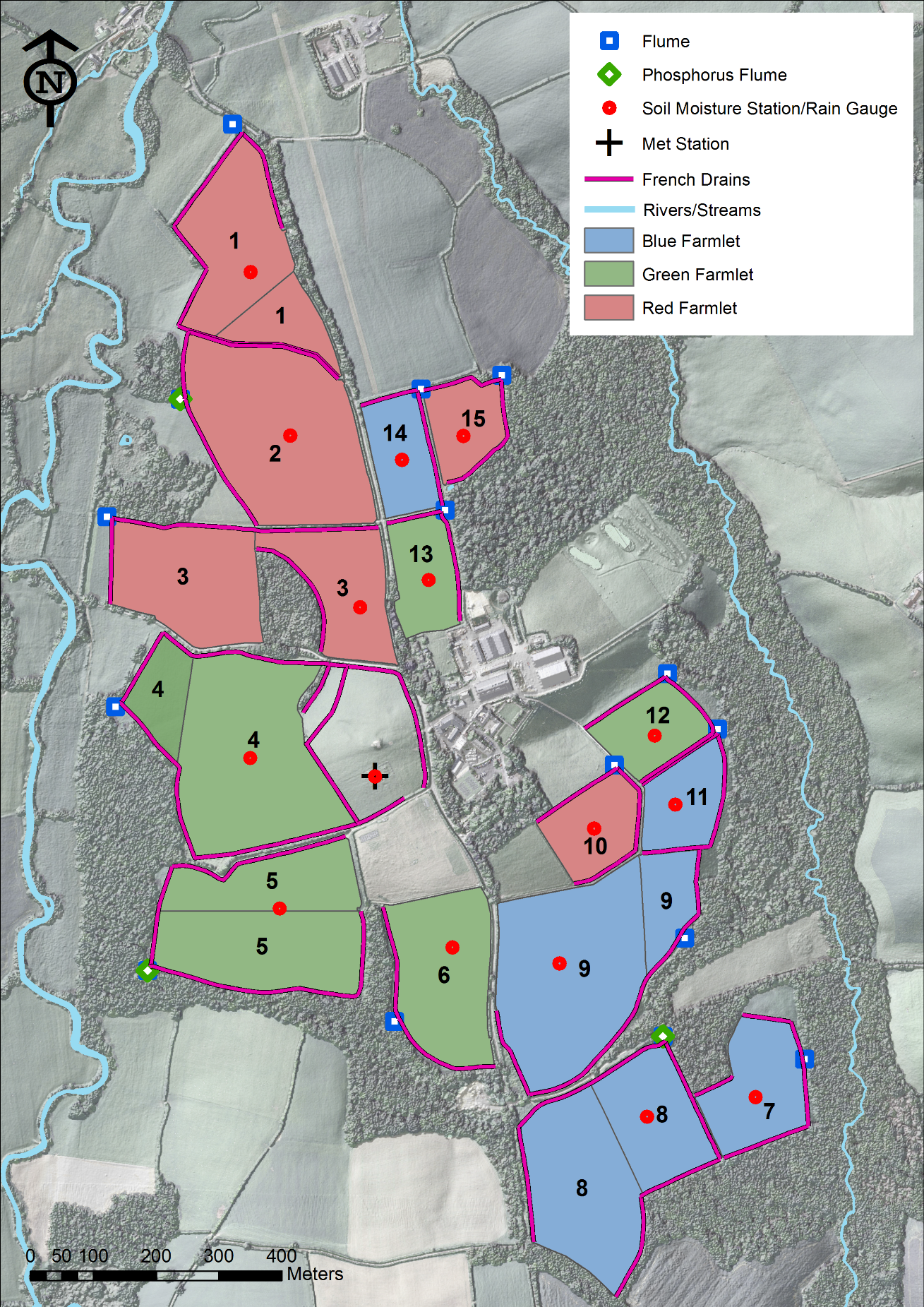


Figure S1. The North Wyke Farm Platform with Red, Green and Blue farmlets (bounded at the edges by French drains) shown, along with the locations of the Soil Moisture Stations and Rain Gauges (red circles) and Flume Laboratories (blue squares). The three Flume Laboratories also capable of recording phosphorus levels are marked (green diamonds). The single on-site meteorological station is marked with a black cross.


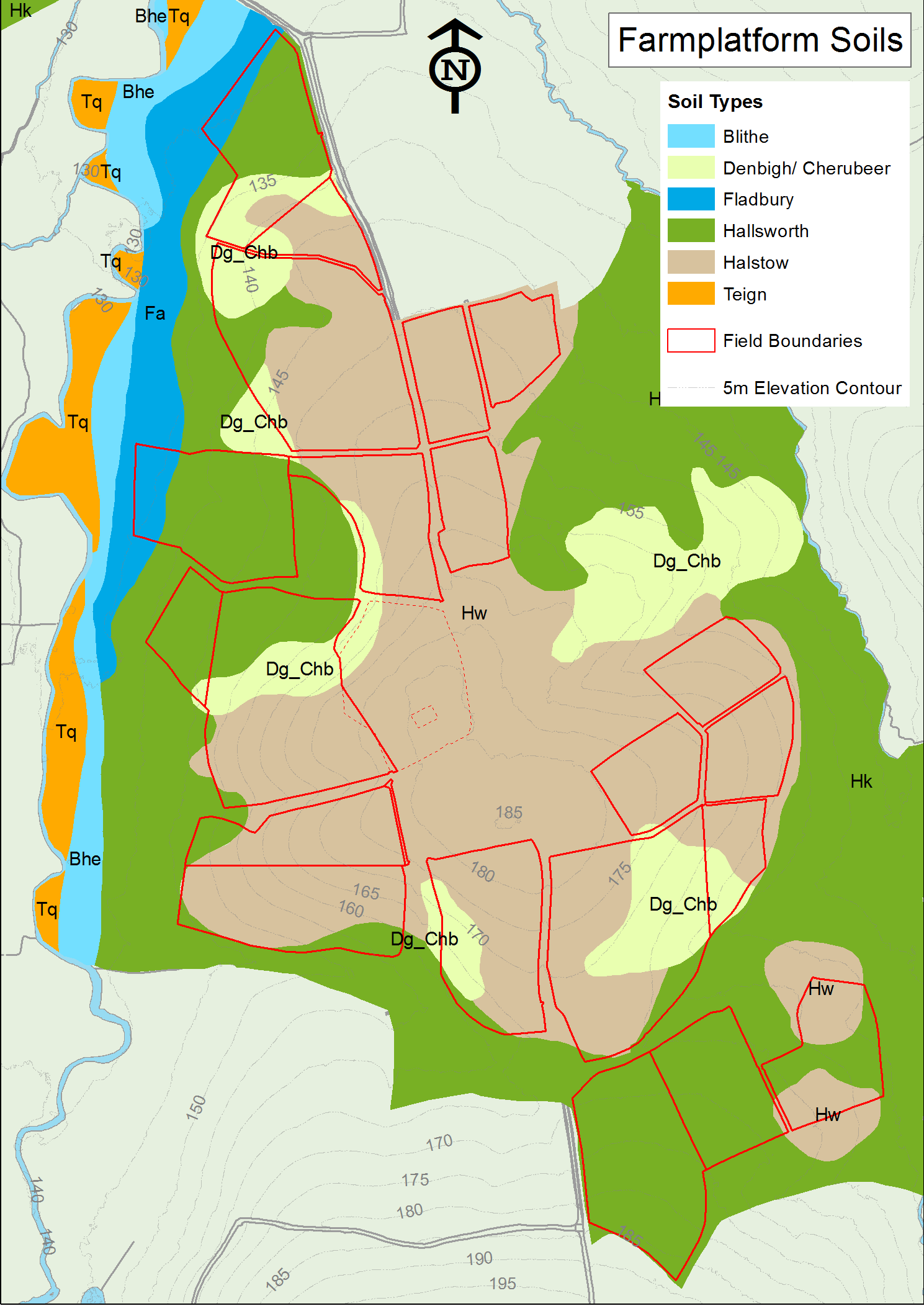


Farm Platform Soils

**Figure S2.** The North Wyke Farm Platform with the field boundaries overlaid on a map of the principal soil series, along with the 5 m contour lines. Blithe (Fluvisol); Denbigh/Cherubeer (Dystric Cambisol); Fladbury (Fluvisol); Hallsworth (Dystric Gleysol); Halstow (Gleyic Cambisol); Teign (Eutric Fluvisol)


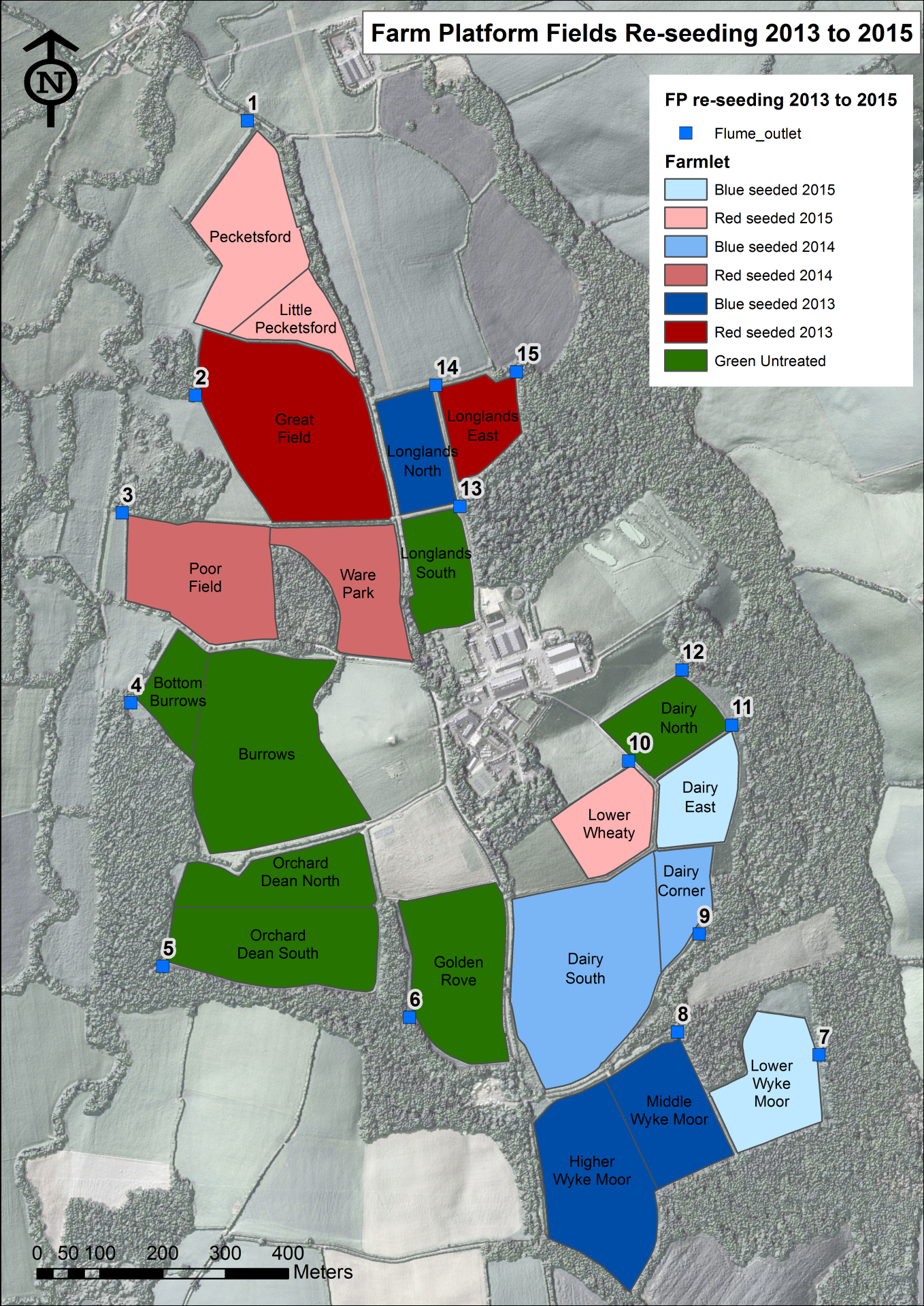


Figure S3. The North Wyke Farm Platform with the re-seeding schedule for the Red and Blue fields in 2013 to 2015 shown. Flume outlets are indicated (blue squares) along with the corresponding catchment numbers. The Green control fields remain undisturbed as long-term permanent pastures.


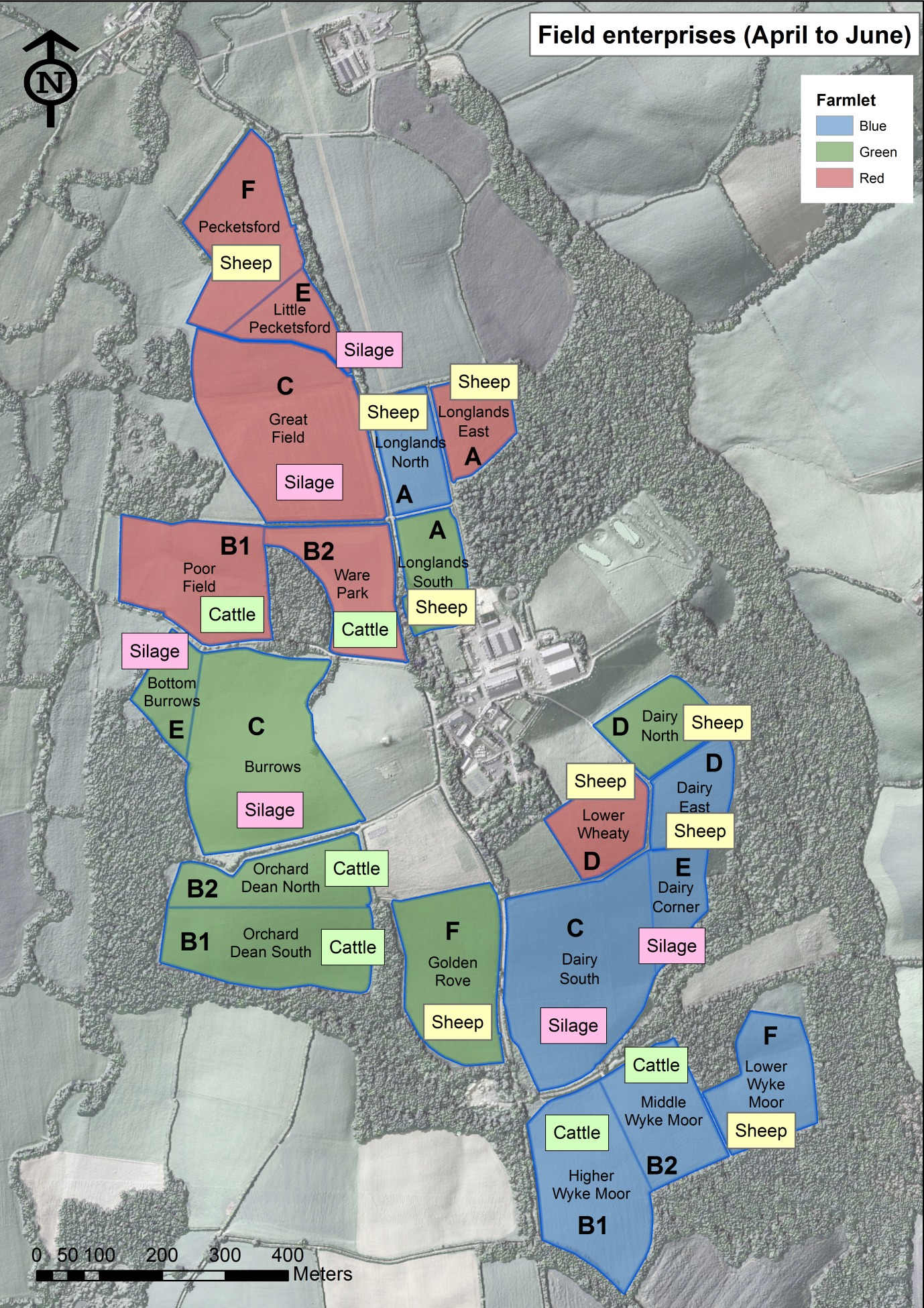


**Figure S4.** The North Wyke Farm Platform with groups of fields (A, B1, B2, C, D, E and F) shown as ‘enterprise triplets’ which are planned to have similar management. Here we show a generalised plan for April to June.
